# Virtual tabletop simulations for primary care pandemic preparedness and response

**DOI:** 10.1136/bmjstel-2020-000854

**Published:** 2021-04-13

**Authors:** Marlot Johanna Blaak, Raad Fadaak, Jan M Davies, Nicole Pinto, John Conly, Myles Leslie

**Affiliations:** 1 W21C Research and Innovation Centre, University of Calgary, Calgary, Alberta, Canada; 2 School of Public Policy, University of Calgary, Calgary, Alberta, Canada; 3 Department of Anesthesiology, Perioperative and Pain Medicine, University of Calgary Cumming School of Medicine, Calgary, Alberta, Canada; 4 Departments of Medicine, Microbiology, Immunology and Infectious Diseases, University of Calgary Cumming School of Medicine, Calgary, Alberta, Canada; 5 Infection Prevention and Control, Alberta Health Services, Calgary, Alberta, Canada; 6 O'Brien Institute for Public Health, University of Calgary, Calgary, Alberta, Canada

**Keywords:** COVID-19, simulation in healthcare, primary care redesign, quality improvement, human factors

## Abstract

**Introduction:**

The COVID-19 pandemic prompted widescale use of clinical simulations to improve procedures and practices. We outline our deployment of a virtual tabletop simulation (TTS) method in primary care (PC) clinics across Alberta, Canada. We summarise the quality and safety improvements from this method and report end users’ perspectives on key elements.

**Methods:**

Our virtual TTS used teleconferencing software alongside digital whiteboards to walk clinic stakeholders through patient scenarios. Participants reviewed and rehearsed their workflows and care practices. The goal was for staff to take ownership over gaps and codesigned solutions. After simulation sessions, follow-up interviews were conducted to collect feedback.

**Results:**

These sessions helped PC staff identify and codesign solutions for clinical hazards and threats. These included the flow of patients through clinics, communications, redesignation of physical spaces, and adaptation of guidance for cleaning and personal protective equipment use. End users reported sessions provided neutral spaces to discuss practice changes and built confidence in delivering safe care during the pandemic.

**Discussion:**

TTS has not been extensively deployed to improve clinical practice in outpatient environments. We show how virtual TTS can bridge gaps between knowledge and practice by offering a guided space to rehearse clinical changes. We show that virtual TTS can be used in multiple contexts to help identify hazards, improve safety and build confidence in professional teams adapting to rapid changes in both policies and practices. While our sessions were conducted in Alberta, our results suggest this method may be deployed in other contexts, including low-resource settings.

## Introduction

COVID-19 has strained healthcare systems globally.^1^ The pandemic’s sudden emergence led to rapid deployment of ‘just-in-time’ preparedness and response strategies aimed at addressing the immediate needs of healthcare systems adapting to uncertain conditions. One such strategy increased the use of clinical simulation to rehearse ‘novel workflows, protocols, and [adaptation] to rapid changes to practice and care delivery’.^2^ This paper reports on the deployment of virtual tabletop simulations (TTS) to identify infection prevention and control (IPC) hazards and threats in primary care (PC) clinics across the province of Alberta, Canada. We summarise the specific quality and safety improvements that followed from the use of this novel TTS method and also report end users’ perspectives on key methodological elements.

In Alberta, COVID-19 pandemic simulations initially prioritised acute care settings, focusing on emergency departments and critical care units. This prioritisation was based on an assumption that acute care facilities would experience the largest and most immediate patient surges.^3^ Led by personnel from Alberta Health Services (AHS)—the agency responsible for delivering acute care in the province—over 400 in situ clinical simulations were delivered to these settings within 3 months of the province’s first identified case of COVID-19.^2^

Outside the acute care system, however, there was neither a capacity to deliver simulations nor familiarity among community-based clinicians with their methods and aims. As independent fee-for-service contractors to the provincial ministry of health, Alberta’s PC physicians fall outside AHS’ purview and thus the reach of AHS-led simulations. Despite a growing need for guidance on how to deal with the ‘seismic effect’^4^ of COVID-19 on family medicine practice, our research team observed PC physicians struggling to adjust to constantly evolving IPC guidance. As elsewhere in Canada, there were few resources appropriately adapted to PC applications and those that existed were difficult to find.^4^ In our role as ‘situated interveners’,^5^ our research team acted on these observations and leveraged our relationships with acute care-based experts in simulation and IPC, as well as leaders in the PC community.^6^ This paper describes how our interdisciplinary team of human factors (HF), IPC and sociology experts rapidly prototyped and scaled an innovative method to deliver TTS—first in person, and then virtually—to PC clinics across Alberta.

This prototyping and scaling work relied on theory that assumes ‘latent conditions’^7^ form a combination of hazards that drive threats in clinical operations. Hazards are inherent in the physical and social structures of a clinical space. They are the unrealised dangers built into the design and functioning of systems. Threats, in contrast, are inherent in operational processes and activities. They are hazards made active as tasks or operations are performed, or system vulnerabilities are made manifest. The TTS sessions we describe here were designed so that, in the course of rehearsing novel activities, IPC hazards and threats inherent in the work of PC clinics dealing with SARS-CoV-2-positive patients could be identified. Our aim was to make latent conditions in PC clinics actionable.

We begin with a detailed description of the PC-focused TTS sessions, which our team prototyped, piloted and scaled over the course of the pandemic. Specifically, we describe the preparation, facilitation and debriefing components of the method. We next summarise the quality and safety improvement outcomes from the simulation sessions. We then present the results of a thematic analysis of follow-up interviews focused on end users’ experiences of the novel method. Our analysis shows that end users found the method valuable, particularly in its balance between facilitative codesign and authoritative expertise. We conclude by highlighting the value of virtual TTS to improve IPC policy implementation in PC settings. We suggest this underutilised method may be of value to multiple user groups to improve pandemic preparedness and response.

## Methods

Our research team conducted 20 TTS with PC teams across Alberta from April 2020 to April 2021. A total of 163 PC team members attended these sessions (110 participants, 53 session observers), and a sample of 10 participants participated in follow-up interviews. Of the PC clinics that participated, 9 were rural and 11 were urban. These numbers also include urgent care clinics that functioned both as primary and acute care centres in rural settings.

The first two TTS were completed in person, with the use of a printed floor plan and tokens, and facilitated by an HF-qualified moderator (MJB), with an additional research team member taking notes on a whiteboard. As the pandemic progressed and inperson meetings were curtailed by public health measures, we transitioned to virtual sessions that used teleconferencing and interactive whiteboard software to provide similar interactivity. These virtual sessions were facilitated by the same moderator and supported by a medical advisor with a combination of IPC and HF experience (JMD). Participants from both inperson and virtual TTS sessions were included in our analyses.

### Virtual TTS preparation

**Figure 1:**
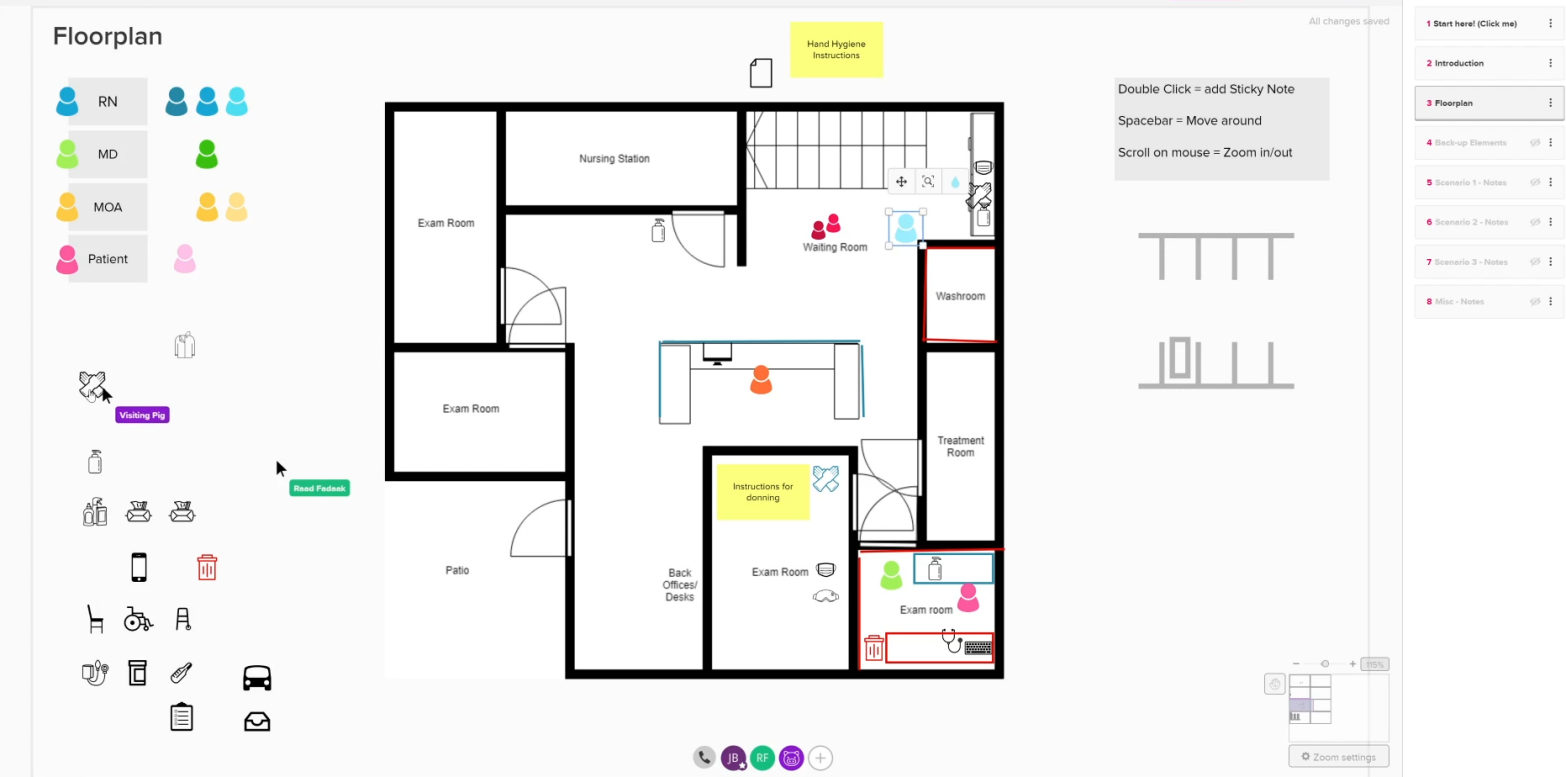
An example of an active MURAL virtual simulation space. The base layer is a clinical floor plan provided by the clinic staff. The coloured tokens represent staff and patient locations, and icons are used to mark locations and placement of personal protective equipment and other supplies. MD, Doctor of Medicine; MOA, Medical Office Assistant; RN, Registered Nurse.

After making initial contact with an interested PC clinic, our team shared a two-page document outlining the value proposition, methodology and action points of an IPC-focused TTS in the pandemic context (online supplemental appendix 1). Clinics that wished to proceed were asked to identify six to eight participants (see the Participants section) and to share a floor plan of their space with the research team. This was entered into the MURAL digital whiteboard software, creating a virtual workspace with a two-dimensional map representing the physical dimensions and layout of the PC clinic (figure 1).

10.1136/bmjstel-2020-000854.supp1 Supplementary data

Taking an engaged scholarship approach,^8 9^ our team worked with PC clinic personnel to coidentify key problematic scenarios that would be most likely to highlight the structures and processes in which IPC hazards and threats might lie. Our approach was based on HF best practices for codesigning and evaluating health systems alongside end users.^10^ Scenarios codeveloped with clinics covered a range of different clinical functions and procedures, such as review of COVID-19 patient screening; interviewing and examining patients with COVID-19 with caregivers and/or family members present; and providing care to COVID-19 presumptive patients with influenza-like illness (ILI) complaints. In addition to these codesigned clinic-specific scenarios, our team deployed a standardised low-probability scenario, which focused on an acutely unwell, rapidly decompensating patient. This improbable scenario was aimed at eliciting imaginative engagement in a situation in which emergency medical services personnel—from outside the usual PC team—became involved.

Before the TTS, an email with step-by-step instructions was sent to all participating clinic staff (online supplemental appendix 2), who were requested to complete and return an informed consent form as part of the research process. The email included Zoom teleconferencing and MURAL whiteboard links, with participants instructed to have both opened, in separate windows, as the session began. This was to facilitate both engagement with the floor plan and active conversation.

10.1136/bmjstel-2020-000854.supp2 Supplementary data

### Participants

As part of the contact and intake process, the moderator encouraged a variety of stakeholders to attend and participate in the sessions as a way to improve cross-team understandings of roles, promote cohesion and improve buy-in for ownership of developed solutions. Within these recommendations, PC clinics nominated key personnel to participate in TTS sessions. These included clinic personnel who were involved in either direct patient care or the creation and implementation of policies and procedures within or across clinics. Physicians, registered nurses, operations coordinators, medical office administrators and clinic managers all participated.

In an effort to balance representation from across the clinic with conversational efficiency, participation was capped at eight, with a minimum of four participants required for optimal interactivity and exchange. In addition, an unlimited number of non-participant observers could also attend the sessions. Observers included clinic administrators, members from the clinic who were not selected to participate, healthcare staff from other clinics and members of our research team. Observers were asked to refrain from participating in the sessions unless the moderator or other participants asked for feedback during scenarios or the debrief. This was planned to allow participants to be able to elaborate on their thinking and decision making without interruption.

### TTS sessions

Sessions lasted for 2–3 hours. Participants were first invited to familiarise themselves with the MURAL workspace by using a purpose-built introductory page. This page allowed them to experiment with icons, tokens and other whiteboard functions in a neutral space. The moderator then began each session by providing an overview of the TTS objectives that emphasised the neutral, non-evaluative nature of the session and highlighted it as an opportunity to identify and codevelop solutions to hazards and threats. This set up a confidential, mutually respectful and quality improvement (QI) focused environment to work in. Once participants were comfortable with the MURAL workspace, the moderator introduced the next page that showed both the simulation agenda and the learning objectives for the session.

The moderator and the medical advisor then guided the participants through the preselected clinical scenarios, asking them to move avatars through the MURAL whiteboard space and stopping at critical junctures to ask IPC-focused questions. Our approach here relied on the principles of ‘cognitive walkthroughs’, a usability evaluation method often used in the field of HF engineering and user design.^11^ Specifically, participants imaginatively enacted COVID-19 care delivery processes inside a representation of the physical structures of their clinic and with the clinic’s policies in mind. These rehearsals allowed participants to talk through their thinking, actions and clinical item/team-mate interactions, and thus to actively identify hazards and threats with guidance from the moderator and the medical advisor. The goal was to enable participants to identify and acknowledge these hazards themselves, and to take ownership over both the gaps and the codesigned solutions.

Both the moderator and the medical advisor were well versed in AHS and local IPC policies and best practices. As such, they acted both as facilitators and consultants. In this way, the hazards and threats identified during sessions led to solutions codesigned by the participants with moderator and medical advisor input. Examples here included the designation of ‘dirty’ (ie, presumptively contaminated) examination rooms or the placement of hand hygiene stations. In instances where solutions were unclear, or disagreements about changes to structure or process occurred, the moderator facilitated conversations focused on achieving workable consensus.

#### Debrief and recommendations

A minimum of 20 minutes was reserved at the end of each session to allow participants to ask any lingering questions or cover discussion points. To conclude the session, the moderator summarised key codesigned solutions and recommendations, ensuring these were converted into action points for those attending the TTS. In the 3 days following the session, the moderator and up to two assistants, as well as the medical advisor and other research team members, would create a summary document report on clinic-specific recommended changes and/or suggestions that arose from the session. This report was generated out of a rapid analysis of the discussions and codesigned solutions presented during the session. The moderator and the medical advisor would ensure that any codesigned solutions were in alignment with IPC guidance provided by local health authorities. As part of checking solutions, the moderator and the medical advisor had access to AHS-IPC experts and our research team’s expert in infectious diseases and IPC (JC).

Additional guidance from these discussions came to be included in these reports, which were returned to clinic staff as a comprehensive reference document to assist in making appropriate changes in structure, policies and practices. The content from clinic-specific reports was then aggregated and de-identified to produce a ‘key learnings’ document that identified common hazards, IPC considerations and best practices for patient management.^12^ This document was shared with and distributed by the provincial medical association and AHS.

### Follow-up interviews to examine end users’ perspectives

Following the simulations, research team members with expertise in the sociology of QI and semistructured interviewing (RF and ML) conducted follow-up interviews (n=10) with selected session participants. Ethnographic interviews are an accepted method for gathering perspectives on QI interventions in outpatient healthcare contexts.^13^ The interviews were conducted between June and August 2020 and lasted 30–45 min. The interviewers used this opportunity to gather TTS participants’ specific feedback regarding the session they had participated in, and eliciting perspectives on what participants believed to be the critical elements of this novel method. Thus, the first portion of the interviews targeted participants’ experiences of the virtual delivery method, the level of preparedness and facilitation approach of the moderator, and the relevance of recommendations. This feedback was de-identified and then relayed from the interviewers to the moderator and the medical advisor in real time to further hone this method.

The second portion of the interviews then focused on key elements in the method as experienced by participants. These qualitative data, aggregated from across the interviews, were analysed to identify not just the benefits that participants associated with the TTS sessions, but the key elements of the method as put into practice by our research team. Our analysis used coded thematic analysis,^14^ conducted in MAXQDA software (V.20.2.2, 2020), with coauthors RF, NP and ML identifying codes and cross-checking one another’s findings. We present a summary of these key benefits and elements from the end user’s perspectives.

## Results

### Virtual TTS sessions: quality and safety improvements

Although many participating clinics had designed and implemented new standard operating procedures (SOPs) to deal with COVID-19, these had often been developed without consideration of the physical and social structures related to clinical processes. Of the 20 participating clinics, the majority (n=13) had drafted SOPs but had not fully communicated them to the staff. Clinical SOPs were not comprehensive and drew on the limited guidance available to the specifics of primary care IPC.^15^ Under these circumstances the TTS sessions became forums for communication roll-out and consensus building—as they were focused on rehearsing scenarios adapted to the participants’ work contexts. These were spaces where new or emerging SOPs could be worked out between staff members and clinic stakeholders. Key QI and safety areas that were addressed in these IPC-focused SOPs included the flow of patients in and out of clinics, communications, effective redesignation of physical spaces in clinics, and adaptation of often vague guidance for cleaning and personal protective equipment (PPE) use.

During each of the TTS sessions, our team worked alongside participants to develop structurally (and thus hazard) sensitive protocols for managing patient entry and exit from the clinic, which included the following:

- Monitoring and enforcing clinic-specific mandates for hand hygiene and mask donning as patients entered.
- Ensuring patients were individually given masks rather than allowed to take them from a box and risk contamination.
- Rescheduling patients who screened positive for COVID-19 symptoms.
- Scheduling blocks of time exclusively devoted to patients with COVID-19/ILI so as to avoid possible exposure of other patients.
- Assigning cleaning responsibilities to specific staff as patients left examination rooms.
- Ensuring patients properly disposed of temporary masks on exiting the clinic.

The TTS sessions also provided PC staff with the opportunity to identify and create SOPs around communication challenges, which included the following:

- Implementation of consistent messaging for patient screening.
- Standardisation of COVID-19/ILI notations in the clinic’s electronic medical record system.

Finally, redesigning physical space and the flow of patients, clinicians and samples through the clinic to meet IPC guidance was a major focus of the recommendations we produced after each TTS session. Designating and separating the clinics into ‘dirty’ and ‘clean’ zones was also mentioned by the moderator and the medical advisor in every TTS. Few participating clinics had designated zones in place or had SOPs that created consistent, practical locations for the examination and isolation of patients, hand hygiene stations, PPE supplies, and essential medical supplies that should be accessible when working with patients with COVID-19. Working alongside the PC teams, the moderator and the medical advisor guided participants in every session to refine SOPs related to physical space usage:

- Redeployed parking lots and patients’ cars as call-in waiting rooms.
- Redesigned waiting rooms to achieve distancing and improve flow.
- Standardised approaches to completing chart work either in a ‘dirty’ zone as an examination finished or later in a ‘cold’ zone.
- Introduced appropriate signage and clear visual indicators about which zone a staff member was in, what sort of activity ought to occur there and whether the area required cleaning.
- Established standards for the transfer of patient samples between collection points and storage areas in the clinic.

A consistent challenge for the moderator and the medical advisor was a deficit in appropriate or applicable guidance in a range of IPC areas. Specifically, a lack of decontamination and cleaning procedures guidance was a major source of anxiety for many participating clinics. While less concerning to the participants, our team identified and sought to remediate a major gap in the guidance available to PC clinics on the effective and appropriate use of PPE. This gap in guidance contributed to deviations from provincial PPE best practices at every participating clinic, including such examples as double gloving, double masking and uncertainty regarding the frequency of PPE replacement.

During each simulation, we provided PC teams with best practices for environmental cleaning that were based on provincial acute care guidance. While posting donning and doffing checklists for PPE^16^ and establishing a buddy system are both proven strategies to help minimise the risk of self-contamination,^17 18^ these were not part of most PC clinics’ SOPs before the TTS sessions.

### Virtual TTS sessions: end users’ perspectives

The thematic analysis of our semistructured follow-up interviews found that the TTS sessions were seen as effective at both cementing existing IPC practice and introducing new policies and procedures. One PC physician participant described how their TTS had included:

enough detail that we felt very confident in the stuff that we were already doing, but then also there [were] a lot of different blind spots with fairly simple solutions. (Participant 37)

Pleased to leave the session with not just ‘action lists’, but the confidence to implement them, the same participant noted that clinic staff:

all found it to be valuable to some degree, because there’s a lot of practical points that we have applied since. (Participant 37)

In addition to these pragmatic and confidence-building benefits, participants identified a number of key elements to the methodological approach described above. These elements included the facilitative approach used by the moderator and the medical advisor, the pragmatic value of the scenarios, the virtual delivery platform and the cross-system perspective brought by our research team.

The combined contribution of the moderator and the medical advisor was called out as important by one participant, who noted:

[The moderator and clinical advisor] were able to throw other things at [us by], posing other scenarios: *‘What if the person has to go to the washroom now? And how do you do this?’* There was enough foundation that people felt confident, and there was great insight from [the moderator and medical advisor] to be able to ask other questions so that all of the teams left knowing that they benefited from participating. (Participant 41)

Participants noted the importance of both the expertise of our team members and the method’s capacity to flexibly introduce new hazards and threats for consideration. Beyond this, the moderator and the medical advisor had built the conversational foundations for PC personnel to feel safe and confident as they rehearsed and experimented with high-stakes IPC issues.

In addition to the importance of setting the tone of the sessions as collaborative, safe spaces, TTS participants identified the scenarios as key elements in the method’s success. These scenarios were viewed by participants to be relevant and realistic, with one participant noting that:

the scenarios were definitely true to what we are seeing in our clinic. (Participant 38)

This was echoed and expanded on by another participant, who noted that:

…Each one of the [scenarios] was definitely something that we could potentially see at the clinic, and had seen [previously]…[and gave us a chance] to think about, ‘what is the best IPC method for maintaining a safe environment for the patient, as well as staff, and then future patients after [a COVID-19] patient leaves?’ (Participant 37)

Virtual delivery of the sessions was identified as another element in the method’s success. The combined use of Zoom and MURAL was seen as catering to multiple learning styles and delivering an interactive and engaging experience. As one participant indicated:

The [avatar] system worked well to visualize it. So it facilitated different learning strategies for visual learners vs didactic learners, auditory learners. It was helpful in that sense. (Participant 39)

Finally, our focus on deploying a single TTS delivery team across multiple PC clinical sites in the province was seen as an element of the sessions’ success. Our team was able to recognise common challenges and share effective solutions, gaining a cross-jurisdictional perspective that helped to anticipate clinical needs, gaps and hazards ahead of time. As one participant pointed out:

I felt like it was very useful because there were things that the [research] team brought up that we didn’t think of. And that was…because they had experience doing this in other clinics and in other settings so they were able to draw from their experience and shoot different scenarios to us that we hadn’t thought of. (Participant 38)

End users found that sessions provided safe, neutral spaces to discuss and debate SOPs while being guided to identify hazards and threats by facilitator consultants from our research team. The sessions were seen as providing benefits in the form of pragmatic, actionable lists of changes, and the confidence to engage in QI.

## Discussion

Clinical simulations, including tabletop exercises, have been widely used both as effective educational tools^19 20^ and as system-based QI techniques.^2 21^ They have been recognised as an effective method to evaluate and optimise clinical environments, by identifying and remediating latent conditions.^22 23^ Increasingly, TTS has been used to prepare for and respond to epidemics and pandemics, including rehearsing national-level responses to hypothetical disease events.^24–26^ At the health system and clinical levels, the COVID-19 pandemic has further ‘cemented simulation programs as fundamental for any healthcare organization interested in ensuring its workforce can adapt in times of crisis’.^27^

While simulations of all kinds have been deployed as a response to COVID-19,^28^ we are aware of very few examples of delivering clinical simulations to PC environments.^29 30^ In this sense, the detailed TTS methodology we have developed and deployed is both consistent with and an innovation in the broader simulation literature. It is consistent in that it aims to bridge the gap between knowledge and practice by offering a guided space to rehearse and talk through change. However, where the focus of much simulation work is on improving ‘the process of care and patient safety across geographically, organizationally, and clinically diverse hospital settings’,^19^ the TTS method described here is innovative in its targeting of PC clinical practice. With PC identified as central to COVID-19 and future pandemic responses,^21 31 32^ the urgency of bringing the benefits of IPC hazard and threat identification to PC increases.

TTS is a less common, but nonetheless proven, technique in healthcare QI that has been used to uncover and address latent conditions in complex healthcare environments^10 33–35^ and as part of public health preparedness.^36^ As such, our TTS drew on elements from various disciplines to adapt a known method to PC during the response to COVID-19. These simulations revealed critical gaps in IPC preparedness, implementation capacity and clinicians’ knowledge of best practices.

With a broad range of IPC hazards and threats identified and mitigated in novel or changed structures and SOPs by PC teams over the course of the sessions, the case for their effectiveness is clear. Beyond being a path to new, consensus-driven SOPs covering patient flow, communication and the safe retasking of physical space, the TTS sessions provided pragmatic, confidence-building forums for PC staff to understand and implement otherwise vague IPC guidance. In addition to identifying IPC hazards and threats, and addressing these with recommendations delivered to each clinic, our end users identified a number of beneficial elements that might otherwise be obscured in the method’s technical details. The approach taken by the moderator and the medical advisor to co-creating the scenarios and facilitating the TTS sessions was particularly important here.

It is critical to position the moderator and the medical advisor as neither fully authoritative ‘outsiders’ nor locally committed ‘insiders’ to the sessions. Rather, we suggest a carefully nurtured ‘alongsider’ status^6 37^ was central to the creation of the safe spaces, confidence and pragmatic solutions that participants appreciated. On the one hand, these ‘alongsiders’ facilitated open dialogue, and even debate, that led to consensus on SOPs and their operationalisation. On the other, they provided evidence and expertise-based answers to questions. Together these meant they were both facilitators and consultants.

Our facilitation work leveraged an engaged scholarship^8 9^ approach to co-creating PC-relevant TTS scenarios and codesigning solutions that reflected local clinical concerns, priorities and patient flow realities. Central to this consultative role was the use of TTS scenarios that would intentionally stress test clinical teams’ IPC capacity and thinking in the safe rehearsal space. Our team’s view of the broader health system, facilitated by our research, allowed us to connect with additional experts as needed.^6^ Our consultative capacity leveraged our own team’s expertise and the experiences of other healthcare providers across the province working to implement IPC best practices.

The TTS method as described here and delivered to Albertan PC teams sought to balance ‘alongsider’ skills—the empathetic management of virtual sessions that were responsive to and codeveloped from local priorities—with outside expertise. This consultative expertise and authority drew on empathy and a depth of knowledge in HF techniques and the practical application of often vague IPC guidance, as well as cross-cutting awareness of Alberta’s health system.

## Limitations

### Methodological challenges

While this intervention was largely viewed as successful in supporting PC teams during COVID-19, there were a number of challenges to overcome in piloting these simulations.

The transition from inperson to virtual sessions presented a unique set of problems. End users were required to have stable, broadband internet connections, a particular challenge in rural settings. They were also required to quickly learn and adapt to the novel technology of MURAL, with the moderator taking time to help them become familiar with the interface. Ensuring all participants felt comfortable moving tokens and avatars around the floor plan took upwards of 5 min, which occasionally delayed the start of the session. In rare cases, end users could not connect to the MURAL space, or could not use both Zoom and MURAL simultaneously.

The virtual format posed a challenge in developing rapport between moderator and session attendees. In some cases, participants were reluctant to share their video and/or contribute to the session. In other cases, end users would talk over one another, which interrupted the flow of discussion. They often did not understand the format of the simulation until the session had begun, which required the moderator to repeat instructions or provide additional clarification to attendees. In rare instances, end users were suddenly disconnected from the session or had to take an urgent, patient-related phone call.

### Evaluation limitations

Our evaluation component relied on anecdotal testimonies of non-randomised participants. In addition, our interviewers, occassionally also attended the simulation sessions. This introduced selection, confirmation and recall bias in the questioning and participants’ feedback. Further, no quantitative measures or surveys were provided as part of the follow-up evaluation process.

## Further directions

While TTS are increasingly used in healthcare settings and disaster preparedness, they have not been extensively deployed in pandemic contexts to improve clinical practice in outpatient settings. Our results suggest that virtual TTS can be used in multiple contexts to help identify hazards, improve safety and build confidence in professional teams who are adapting to rapid changes in both policies and practice. While our application was restricted to PC in Alberta, this low-cost, high-impact method may be deployed in other settings globally, including low-income and middle-income countries. The virtual delivery of TTS may also provide value in other disaster responses and humanitarian emergencies, both reactively, as in our example or, preferably, proactively.

**What is already known on this subject**

- The COVID-19 pandemic has greatly strained the health systems and further established simulation as a critical tool in assisting healthcare workers to adapt to the rapid pace of change.
- Tabletop simulations (TTS) are a widely accepted effective tool for education and quality improvement in healthcare, but have been underused in primary care.

**What this study adds**

- This paper describes a novel, virtual application of TTS to support primary care clinicians as they adapt their infection prevention and control practices to the pandemic.
- We highlight our interdisciplinary team’s approach which used non-evaluative facilitation and provided evidence-based recommendations.
- Our method assisted primary care personnel to codesign solutions to latent hazards and threats in their clinics.
- Virtual TTS offer a low-cost and effective means to support front-line pandemic responders in a variety of contexts, including those in low-income and middle-income countries.

## Data Availability

Additional data are not publicly available, as they contain identifiable clinic, provider and/or patient information and are protected under our ethics agreement with the University of Calgary’s Conjoint Health Research Ethics Board. For more information, contact chreb@ucalgary.ca.
