# Supplementary material for: Virtual tabletop simulations for primary care pandemic preparedness and response
Source: BMJ Simul Technol Enhanc Learn. 2021 Apr 13;7(6):487–93. doi: 10.1136/bmjstel-2020-000854 (PMC8327409; doi:10.1136/bmjstel-2020-000854)
Supplement: Supplementary data [file bmjstel-2020-000854supp001.pdf]

Policy Implementation and Communication Lessons from Alberta's Acute and Primary Care Environments During the COVID-19 Response -- Principal Investigators: Dr. Myles Leslie and Dr. John Conly

COVID-19 Primary Care Tabletop Simulations

Our federally funded project aims to **examine and support Alberta's response to COVID-19**. We are conducting interviews and site visits at public health, acute and primary care, and community organizations to understand the flow of communications and preparations. We are proposing to partner with primary care clinics to carry out a particular kind of site visit, involving guided **tabletop simulations** and **interviews**. The simulations will be aimed at providing clinicians the opportunity to **review, rehearse, and improve their response to COVID-19** by systematically reviewing policies and protocols, and adapting them to local conditions. Interviews will focus on the broader context of communications related to the outbreak response.

Our goals for the simulations and interviews are to:

› Evaluate the structures (policies, pathways, guidelines, processes) that clinics have in place to respond to a suspected COVID-19 case.
› Assess system effectiveness and efficiency – alongside primary care staff – and co-design concise and clear recommendations for clinics to reduce errors and optimize processes.
› Understand how the recommendations integrate into the communications and preparations clinicians have been receiving to date.
› Aggregate learnings to apply to operations elsewhere in Alberta's Primary Care environments.

Tabletop simulations are a novel, low-resource tool to evaluate system elements and clinic preparedness. These simulations will take place **in clinics** (in an available meeting room) or **virtually** with clinic stakeholders and a facilitator from our team.

Data Collection: Audio capturing of tabletop sessions and individual interviews, photographic capturing of the tabletop session and relevant clinical spaces, notes taken by the observers.

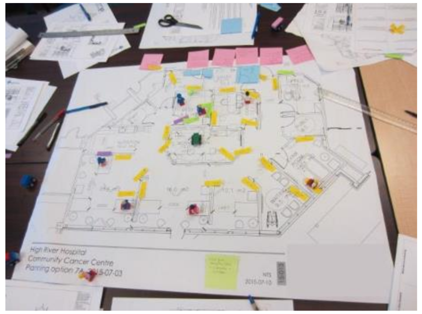

For questions or further information contact:
Johanna Blaak | W21C Human Factors Research Associate
marlot.blaak@ucalgary.ca
403.200.4252

Alberta Health Services

This work will be done with support from the **AHS Human Factors team**

Session Procedure
In preparation, our team will coordinate logistics with the clinic's leadership and select which scenario(s) to simulate.

The **session** will start with an introduction of the project and tabletop simulation. All participants will be asked to sign an informed consent form.

Our team will facilitate clinic stakeholders as they simulate the arrival and care of a patient. This will be done on top of a printed floor plan of the clinic. Any issues or topics that need to be discussed will flagged for de-briefing sessions.

During the **debriefing,** we will analyze and discuss flagged items to co-create action points and recommendations for patient and process management.

Follow up **interviews** will then be conducted as appropriate on site, or later by phone.

Here's what we'd like your staff to do in preparation for the tabletop session:

In Person Sessions

The value of in-person settings is to create a dynamic and collaborative co-working space. During these we will observe proper IPC protocols.

1. **Coordinate** internally, and with our team, to **set up a session time** and **reserve a room for two hours**;
   › The room should accommodate 5-10 people with enough room for social distancing.

2. **Recruit 6-8 participants** from the clinic to join the session during the specified time/day;
   › Participants should be selected from key personnel who would most likely be involved in COVID-relevant care or patient management;
   › Please **send the list of recruited participants** to our team, along with their roles/titles;
   › Additional observers are permitted if they wish, with a maximum of 10 total staff members present during the session.

3. **Share, if available, a clinic floorplan** with our team;
   › We will bring printouts of this floorplan to use in the simulation exercise.

4. **Select and adapt patient scenarios** (*see on the right*) at least one day prior to the session.
   › Please **return these to our team** so we can generate a plan for the simulation.

Virtual Sessions

We want to acknowledge that barriers in collaboration may exist during virtual sessions, by using the right platforms and procedures we can still produce valuable insights. Virtual sessions are conducted using the online platform Mural and Zoom teleconferencing software.

1. **Coordinate** internally, and with our team, to **set up a two hours session time.**

2. **Recruit 6-10 participants** from the clinic to join the session during the specified time/day;
   › Participants should be selected from key personnel who would most likely be involved in COVID-relevant care or patient management;
   › Please **send the list of recruited participants** to our team, along with their roles/titles and e-mail;
   › Additional observers are permitted if they wish, any observers should be announced to the study team.
   › All participants should have access to a computer with audio and mic.

3. **Share a clinic floorplan** with our team;
   › The digital space will be set-up using this floorplan.

4. **Select and adapt patient scenarios** (*see on the right*) at least one day prior to the session.
   › Please **return these to our team** so we can generate a plan for the simulation.

Patient Scenarios

Scenarios can be adapted to reflect the clinic's expected encounters, is most concerned about, or has already identified as a COVID-19 related challenge.

Patient call screening;
› Making appointments for patients with ILI symptoms,
› Fielding questions about COVID testing/care options.

Patient tele-health consultation;
› Using new billing codes.
› Set-up of tele health infrastructure.

Patient presenting in-person with presumptive/confirmed COVID-19;
› Patient flow through clinic,
› Checking-in and triaging individual patients or families,
› Reviewing PPE use,
› Managing contamination and cleaning.

Walk-in patients with ILI symptoms;
› Managing unexpected demand,
› Identify opportunities for task shifting.

Managing acutely unwell patients with presumptive/confirmed COVID-19;
› Coordinating emergency response in clinic,
› 911 Call and EMS presence.
