# Supplementary material for: Virtual tabletop simulations for primary care pandemic preparedness and response
Source: BMJ Simul Technol Enhanc Learn. 2021 Apr 13;7(6):487–93. doi: 10.1136/bmjstel-2020-000854 (PMC8327409; doi:10.1136/bmjstel-2020-000854)
Supplement: Supplementary data [file bmjstel-2020-000854supp002.pdf]

Hello, everyone!

Thank you for agreeing to participate in tomorrow's Virtual Tabletop Simulation session – **DATE/TIME**.

This is a novel, low-resource tool to assess system elements and clinic preparedness in response to COVID-19. Our goal is to work through a set of scenarios together and look at the safety and effectiveness of your clinic, and co-design concise and clear recommendations to optimize the structure and processes you have in place.

Before the session:

› Please find the attached Consent Form. Take a moment to read it and send back a signed copy as soon as you can (this can be after the simulation, showing up for the session will be used as implied consent in this case).

Joining the session:

› STEP #1: Please click on the following link to join our Zoom meeting. Your microphone will be muted automatically on entering the meeting.

ZOOM DETAILS HERE

› STEP #2: Please click on the following link to join MURAL. You will land on a page with some instructions on how to navigate Mural. Feel free to practice.

MURAL LINK HERE

During the session:

› The moderator will give an introduction into the activity and platform, and lead the team through the different scenarios.

› You can use sticky notes to record important points. On the Mural floor plan, there will be a box of equipment, e.g., a wheelchair, and you can move each piece of equipment to its appropriate location in the Clinic, if needed.

› At the end of the session, the moderator will debrief all participants. During the debriefing, we will analyze and discuss flagged items to co-define action points and co-create recommendations for any structural and patient management process improvements.

› After the session you will receive a summary and recommendations document based on our discussion.

Looking forward to meeting all of you! In the meantime, please don't hesitate to contact me if you have any questions.

Best,
